# Surgical career choices of medical students in China: does gender bias play a role?

**DOI:** 10.1186/s12909-022-03453-0

**Published:** 2022-05-17

**Authors:** Liangru Zhou, Bingjie Liu, Wenqi Fu, Wenhao Wu, Yan Wang, Peiyan Ju, Xin Zhang, Guoxiang Liu

**Affiliations:** School of Health Management, Harbin Medical University, 157 Baojian Road, Nangang District, Harbin, China

**Keywords:** Gender bias, Surgical career, Medical student, Career choice

## Abstract

**Background:**

Gender bias in career choices has always been a matter of great concern, including in the field of medicine. This study reports on the current situation in this regard in China, including the reasons for Chinese medical students’ willingness to engage in surgical careers; investigates their accounts of gender bias; and analyzes the effect of gender bias on their surgical career choices.

**Methods:**

This study invited medical students from Harbin Medical University to fill out a non-mandatory questionnaire on whether they had witnessed gender bias, their surgical career intentions, and factors influencing their career intentions. A one-way analysis of variance was used to compare the differences between continuous variables. Pearson’s chi-squared test was used to compare the differences between the categorical variables, the Kendall correlation coefficient (tau) was used to assess the correlation between the reasons rankings reported by gender, and a multiple regression analysis was conducted by logit model.

**Results:**

A total of 643 students responded to the questionnaire. Of them, 63.76% expressed a willingness for a surgical career, with “interest” being a key driving factor (73.41%). Almost all respondents (96.27%) answered that there were more male leaders in the surgical departments they had rotated through or had contacted. Only a few respondents reported gender barriers influencing recruitment (32.19%). However, witnessing gender bias (recruitment of male required) was correlated to choice of surgical career (*P* < 0.05). Females were less willing to pursue a career in surgery if they had witnessed gender barriers in surgical recruitment. Male dominance also correlated to the choice of a surgical career (*P* < 0.1). Of the respondents, 53.19% believed that surgery was not suitable for females; among female respondents, this number was 56.12%, higher than for male respondents. When females think that the surgical profession is not suitable for them, it reduces the possibility of their pursuing a career in surgery.

**Conclusion:**

Most medical students were interested in surgical care. Witnessing gender bias decreases females’ willingness to pursue a career in surgery. It is necessary to stimulate medical students’ interest in surgery when formulating strategies to promote surgical career choices, as well as to reduce gender bias in surgery; in this way, females’ surgical careers should be ensured.

## Introduction

Career selection is a complex process that is affected by many factors. Studies have revealed several factors related to career choices, such as prestige and professional interests, controllable lifestyle, and gender [1–4]. Gender in particular plays a very important role in career selection. Gender bias in STEM fields has gradually decreased in recent decades, but it has not been completely eradicated [5], including in the medical field [6].

Female health service providers serve about 5 billion people worldwide, accounting for 70% of the health and social care workforce [7]. However, their role as a driving force in the health care field is often not recognized or valued, and gender inequality is still widespread in health fields. Although females provide most health services, global health leadership is dominated by males, with 69% of global health organizations led by males [6].

In particular, males were long considered more suitable than females for surgical careers, due to traditionally accepted gender norms and stereotypes [6]. One review from Sweden suggests that surgery remains male-dominated, with females making up only 10%–20% of surgeons [8]. Researchers in the Netherlands investigated gender differences in career choices among medical students, and found that males prefer surgical occupations, while females prefer non-surgical occupations [2]. This is supported by a study in the United Kingdom, which shows that most people think surgery is not a welcoming career path for female trainees [9]. Research conducted in South Korea shows differences in choice of medical specialties among medical students of different genders [3]. A systematic review of global context conducted in 2018 also confirmed that gender bias prevents female medical students from entering the surgical profession [10]. In the United States, the gender gap in the field of surgery is narrowing, although males still outnumber females by at least a factor of five, except in the subspeciality of plastic surgery, which is reaching gender parity in the U.S. [11].

Gender bias prevents females from entering the surgical field for many reasons. Industry requirements and stereotypes can influence a female’s entry into this field [12]. In addition, the existence of the “old boys club” also prevents females from becoming surgeons [13]; in order to maintain masculine cultural standards and status, members of the “club” form alliances within an informal male social system that extends both inside and outside the organization, excluding females [14]. The World Health Organization reports that the surgical profession is a male domain [6], while females tend to be considered more suitable for nursing and similar jobs. Lack of opportunities for career development are also an important contributing factor. A global study involving 75 countries showed that difficulties faced by females’ in obtaining postgraduate training reduces their likelihood of considering a career in surgery by 60% [15]. Some studies have also pointed out that female medical students are more likely to be reluctant to pursue a career in surgery due to the lack of female role models [16]. Thus, we can see that gender bias in the surgical field limits the possibility of gender equality, negatively impacting health systems and the delivery of quality care [6].

Although there have been many studies on gender inequality in surgery worldwide, there is almost no such literature in China, which has a large medical system. According to the 2019 China Health Statistics Yearbook [17], there were 3.607 million licensed (assistant) physicians in China in 2018, of whom 12.2% were surgeons. The Chinese College of Surgeons’ “Investigation Report on the Practice Status of Chinese Female Surgeons (2019)” revealed that the proportion of female surgeons in China is increasing, but still only comprises 6.04% of Chinese surgeons, and that a majority of them are residents and attending physicians [18]. Therefore, it is necessary to address gender inequality in the surgical field in China. To provide more data in this regard, this study evaluated the impact of gender bias on Chinese medical students’ choice of surgical careers. Furthermore, this study also plans to explore the preferences of this group of surgical professionals and the reasons for those preferences, to clarify what improvements may be needed.

## Methods

### Participants

Students from Harbin Medical University were invited to participate in this study. Students who had undergone clinical internships were expected to have a deeper understanding of the clinical work. Simultaneously, they would have faced professional choices at the graduate level, so the career choices of these students would be more realistic and prudent. Therefore, students in the third year of their medical education and above were selected to answer our questionnaire.

### Data collection

This study designed a questionnaire (see https://www.wjx.cn/) on surgical occupational intentions and gender bias, with both single- and multiple-choice questions. If the respondents had answers other than the given options, they could provide them by filling in the blanks. To improve the questionnaire, a pre-survey was conducted with graduate students who had a more comprehensive understanding of the surgical profession. Data from the pre-survey were not used for data analysis. We collated the comments received and revised the questionnaire accordingly; two researchers then reviewed the questionnaire to ensure its quality before distribution. The instructor showed all participants a QR code linked to the questionnaire through the clinical course. Respondents used their mobile phones to fill out the questionnaire, which took 1–3 min. The questionnaire also collected basic information such as gender, age, residence (rural or urban), and academic major.

The question “Willingness to pursue a surgical career?” was used to investigate the willingness of medical students to choose a career in any of the nine recognized surgical specialties in China (and to specify their preferred ones): general surgery, cardiothoracic surgery, urology, orthopedics, oral and maxillofacial surgery, otorhinolaryngology, plastic surgery, neurosurgery, and children’s surgery. Respondents were also asked to report their reasons for willingness or unwillingness to engage in surgery as a career, using two multiple-choice questions. Students who were willing to pursue a career in surgery were required to report their preferences for surgical departments.

Three questions were used to investigate gender bias: an inclination or prejudice against the gender of a person or group, especially in a way considered to be unfair, that often results in discrimination [6]. The question “Are males that have been rotated in or contacted the surgical department dominant as leaders?” was used to investigate the representation of females in leadership roles [10]. The question “Is the recruitment of males required (or implied required) in surgical recruitment?” was used to investigate gender bias in job search. It comes from graduates who have experienced gender constraints. Finally, the question “Is the surgical profession suitable for females?” was used to gather respondents’ judgments on gender bias. Respondents who reported that females were unsuitable for a career in surgery were asked to give a reason. All respondents answered all three questions about gender bias.

### Data analysis

Analyses were performed using Stata 15.0. One-way analysis of variance was used to compare the differences between continuous variables, and Pearson’s chi-squared test was used to compare the differences between categorical variables. The Kendall correlation coefficient (tau) was used to assess the correlations between the causal rankings reported by gender. When two sortings are exactly the same, tau = 1; when two sortings are completely opposite, tau = -1; when two sortings are randomly generated and completely different, tau = 0. A logit model was used to conduct a multiple regression analysis. Three models were set up for multiple regression: Model 1 only included the personal characteristics of the respondents, Model 2 added gender bias issues based on the personal characteristics of students, and Model 3 added interaction terms based on Model 2. *P* < 0.05 indicates that the results are statistically significant.

## Results

### Response and career choice in surgery

A total of 643 questionnaires were collected in this study, with effective response rates of 100%. The average age of the respondents was 21 years (range: 18–24); of them, 376 were females (58.48%) and 267 were males (41.52%). A total of 205 respondents (31.88%) were from rural areas, and 438 (68.12%) from urban areas. There were 569 (88.49%) respondents from clinical majors, 25 (3.89%) from pediatrics, and 49 (7.62%) from dentistry. The sample demographics are summarized in Table 1.

**Table 1:** Basic characteristics of respondents

Characteristics	Composition/ mean (%/)	Choose surgical career	*P*-value
		**Composition/mean**	
**Total**	643(100%)	410(63.76%)	——
**Age**	21.21(18–24)	21.21(18–24)	*P* = 0.78
**Gender**			*P* = 0.00
Male	267(41.52%)	220(53.66%)	
Female	376(58.48%)	190(46.34%)	
**Residence**			*P* = 0.96
Rural	205(31.88%)	131(31.95%)	
City	438(68.12%)	279(68.05%)	
**Major**			*P* = 0.69
Clinical	436(67.81%)	273(66.59%)	
Clinical 5 + 3	133(20.68%)	90(21.95%)	
Pediatrics	25(3.89%)	17(4.15%)	
Oral cavity	49(7.62%)	30(7.32%)	

Clinical 5 + 3: Clinical undergraduate 5 years + postgraduate 3 years

### Career choice in surgery and reasons cited (sorted)

#### Preference for surgical career and preference among subfields

Overall, 410 people (63.76%) expressed a willingness in pursuing a surgical career. Among them, 220 male respondents accounted for 84.2% of the total number of male respondents and 190 female respondents accounted for 50.53% of the total number of female respondents. There were no statistical differences in the career choices of students by age, residence, or major (*P* > 0.05). Based on these results, different genders gravitated towards statistically different career choices (*P* = 0.000) (Table 1).

**Fig. 1:**
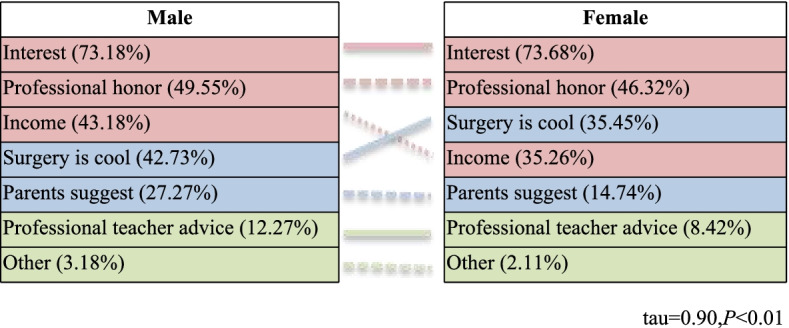
Respondents’ reasons for interest in a surgical career

This study ranked reasons for willingness and unwillingness to pursue a surgical career separately by gender. Figure 1 shows why medical students of different genders were interested in surgical careers. Calculating the similarity of the reasons why male and female respondents wished to pursue a surgical career, the Kendall’s tau value is 0.90, indicating very similar reasons. Among them, “interest” (73.41%) ranked first, followed by “professional honor” (48.05%), “surgery is cool” (41.95%), “income” (39.51%), “parents suggested it” (21.46%), and “professional teacher’s advice” (10.49%) (Fig. 1).

**Fig. 2:**
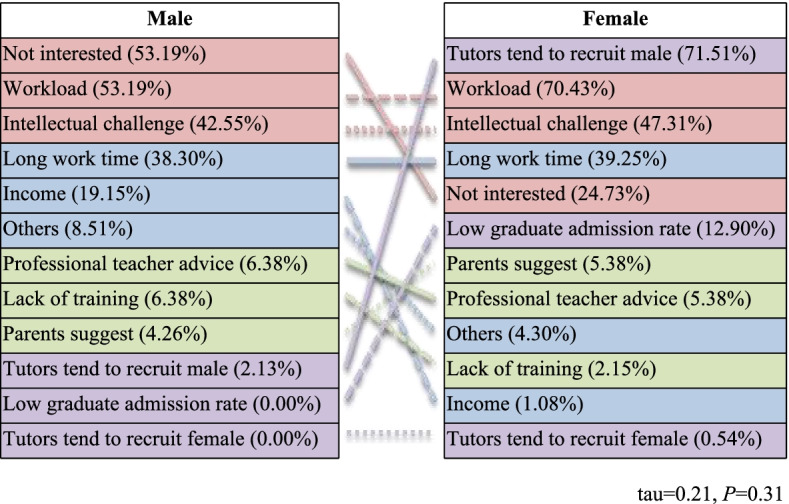
Respondents’ reasons for lack of interest in a surgical career

The tau value of the reasons for unwillingness to pursue a surgical career was 0.24, indicating that the reasons for unwillingness to choose a surgical career were significantly different between male and female respondents (Fig. 2). “Not interested” and “workload” (53.19%) tied as the top reasons why males were unwilling to engage in surgery. The most common choice among females was “tutors tend to recruit males” (71.51%), followed by “workload” (70.43%).

**Fig. 3:**
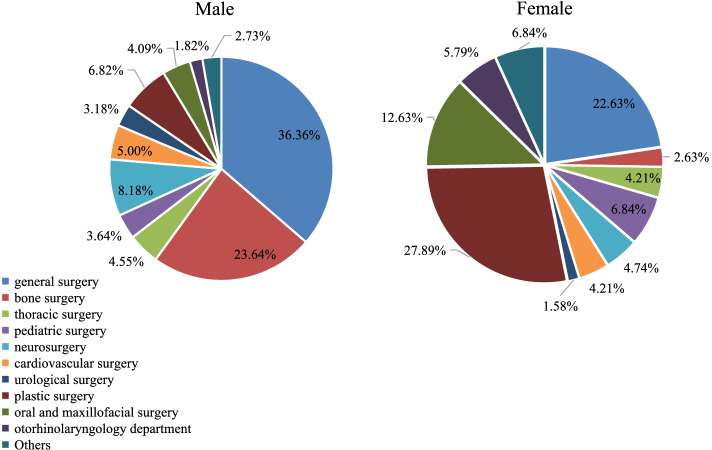
Surgical subfield preference by gender

Medical students of different genders again had different preferences when it came to their choice of surgical department (Fig. 3). Males preferred general surgery (36.36%), orthopedic surgery (23.64%), and neurosurgery (8.18%), while females preferred plastic surgery (27.89%), general surgery (22.63%), and oral and maxillofacial surgery (12.63%).

### Gender bias in surgery

More than 95% of the respondents (both males and females) answered that there were more male leaders in the surgical departments when they were rotated/contacted. In all, 75.66% (202) of males and 62.23% (234) of females believed there were no gender barriers in recruitment. There was no statistical difference in perceived male leadership dominance according to gender (*P* = 0.09); however, there was a statistically significant difference in perceived gender barriers in recruitment (*P* = 0.00). In response to the question “Is a surgical career suitable for females?” 49.06% (131) of male respondents chose “not suitable,” as did 56.12% (211 persons) of females; there was no statistical difference between genders (*P* = 0.07) (Table 2).

**Table 2:** Gender bias by gender

	**Male leadership dominance**			**Gender barriers in recruitment**			**Surgical career suitable for female**		
**Gender**	Yes	No	*P* = 0.09	Yes	No	*P* = 0.00	Suitable	Not suitable	*P* = 0.07
Male	261 (97.75%)	6 (2.25%)		65 (24.34%)	202 (75.66%)		136 (50.94%)	131 (49.06%)	
Female	358 (95.21%)	18 (4.79%)		142 (37.77%)	234 (62.23%)		165 (43.88%)	211 (56.12%)	

**Fig. 4:**
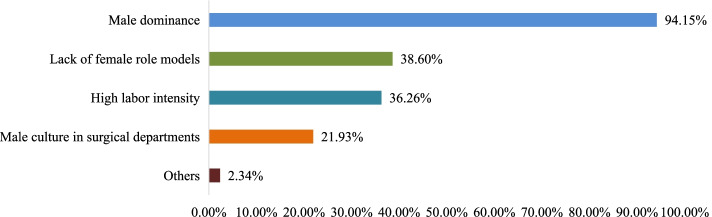
Respondents’ reasons for finding females unsuitable for surgery

The main reason that females were viewed as unsuitable for the surgical profession was the "Male dominance" associated with the profession (94.15%), followed by "Lack of female role models" (38.6%) and “High labor intensity” (36.26%) (Fig. 4). Respondents generally believed that physiological factors, such as physical insufficiency and fertility, would affect the development of females in the field of surgery. In addition, some respondents pointed out society’s expectations of females and the prejudice of mentors as reasons for the view that “females are not suitable for surgical professions.”

### Does gender bias affect surgical career choice?

Among the 619 respondents who reported male leadership dominance, 393 were willing to start a career in surgery, including 215 (54.71%) male respondents and 178 (45.29%) female respondents. Among the 207 respondents who reported gender barriers in recruitment, 72 were unwilling to start a career in surgery, of whom 6 (8.33%) were males and 66 (91.67%) were females. A total of 342 respondents believed that females were not suitable for surgery, including 131 (38.30%) males and 211 (61.70%) females (Table 3).

**Table 3:** Surgical career choice by gender

Bias	Choose surgical career		
	**Yes(row%)**	**No(row%)**	**Total(row%)**
**Male leadership dominance**	393	226	619
Male	215(54.71%)	46(20.35%)	261(42.16%)
Female	178(45.29%)	180(79.65%)	358(57.84%)
*P*-value	0.00		
**Gender barriers in recruitment**	135	72	207
Male	59(43.70%)	6(8.33%)	65(31.40%)
Female	76(56.30%)	66(91.67%)	142(68.60%)
*P*-value	0.00		
**Female are not suitable for surgery**	185	157	342
Male	106(57.3%)	25(15.92%)	131(38.3%)
Female	79(42.7%)	132(84.08%)	211(61.7%)
*P*-value	0.00		

The results of Model 1 indicate that being female was associated with a decrease in willingness to engage in surgical occupations (*P* < 0.01). Model 2, with gender bias variables, shows that perception of gender barriers in recruitment was positively related to an increase in participants’ willingness to engage in a surgical career (*P* = 0.015), while perception of male leadership dominance showed no relationship with it (*P* = 0.068). When a female thought she was unsuitable for a surgical career, her willingness to choose a surgical career reduced. In the results of the interaction effect model, the coefficient of the main effect variable of whether the surgical profession is suitable for females was -0.36, and the coefficient of the interaction term between the suitability of a surgical career for females and gender was -0.88. If females think that a surgical career is not suitable for them, it reduces their likelihood of engaging in one. At the same time, although non-significantly, females showed less willingness to pursue a surgical career if they saw gender barriers in the recruitment process. The average partial effect for female respondents was -0.19, which means that female medical students are 19% less likely to choose surgery as a career than males (Table 4).

**Table 4:** Multivariate analysis for gender bias and surgical career choice

Characteristic	Model 1						Model 2				Model 3			
	coefficient			95%CI		margins	coefficient	95%CI		margins	coefficient	95%CI		margins
Age	-0.15	-0.39			0.08	-0.03	-0.14	-0.39	0.1	-0.03	-0.15	-0.4	0.09	-0.03
Female	-1.54***	-1.92			-1.16	-0.32	-1.65***	-2.11	-1.26	-0.32	-0.98***	-1.58	-0.38	-0.19
Resident	-0.05	-0.42			0.33	-0.01	-0.04	-0.42	0.34	-0.01	-0.05	-0.44	0.34	-0.01
Major														
Clinical 5 + 3	0.09	-0.35			0.53	0.02	0.04	-0.41	0.49	0.01	0.06	-0.4	0.52	0.01
Pediatrics	0.27	-0.64			1.18	0.05	0.43	-0.51	1.36	0.08	0.44	-0.51	1.38	0.08
Oral cavity	-0.04	-0.69			0.61	-0.01	-0.07	-0.78	0.64	-0.01	-0.1	-0.8	0.61	0.02
Bias														
Leader							-1.23	-2.56	0.1	-0.24	-1.08	-2.4	0.23	-0.21
Recruit							0.48**	0.09	0.87	0.09	1.05**	0.13	1.97	0.2
Unsuitable							-0.97***	-1.34	-0.61	-0.19	-0.36	-1.02	-0.29	-0.07
Gender*recruit											-0.73	-1.755	0.29	-0.14
Gender*not suitable											-0.88**	-1.67	-0.1	-0.17
_cons	4.8		-0.27		9.87		7	1.38	12.63		6.45	0.81	12.09	

Leader: Male leadership dominance; Recruit: recruitment of male required (or implied required); Unsuitable: females are not suitable for surgery

^*^* *P* < 0.05.;*** *P* < 0.01

## Discussion

The research results showed that most medical students were interested in pursuing a surgical career (63.76%). This result was higher than the proportion of students interested in surgery reported at Jena, in Germany (25%) [19]. It was also higher than the preference for surgery among Mexican medical students (52.4%) [20]. The method of questioning and nature and phrasing of questions might explain this difference. This study focused on the respondents’ interest in the surgical profession, and surveyed them with a binary yes/no question: “Are you willing to pursue a surgical career?” This centralizes the respondents’ choice to a certain extent, in contrast to other surveys, which usually provided more options besides surgery, possibly obscuring the overall picture to an extent.

Compared to female respondents (50.53%), male respondents (82.40%) were more inclined to choose a surgical profession (*P* < 0.01). “Interest” was a key driver for medical students to pursue a career in surgery (73.41%). It has also been found to be a common factor in the career choices of Syrian [21] and Japanese medical students [22]. Both male and female respondents in this study chose surgical careers for similar reasons, namely interest and professional honor, which differs from the results of a study in Toronto [23], where career choices of male medical students were mainly affected by technical challenges, income, and prestige, while female medical students paid more attention to the availability of internships, job opportunities, and maternity leave. In contrast, the reason why some Chinese medical students chose to pursue a surgical career in this study was that they were “hoping to bring some convenience to their families.” Medical students have more medical expertise than non-medical professionals, and can thus answer health queries for their families and take care of their families’ health better. At the same time, many Chinese medical students, across genders, in this study were unwilling to engage in surgery, for a range of reasons. Male respondents mainly considered their level of interest in surgery and the workload of the surgical department, whereas “tutors are more inclined to recruit males” was the primary reason for female respondents’ reluctance to pursue a career in surgery. Since males have a perceived gender advantage in the field of surgery, they mainly considered interest and labor intensity, both of which are situation-independent factors. Therefore, females who are interested in a surgical career may give up their surgical career due to the gender bias of the tutor.

Although increased female representation in the field of medicine has been well-documented [24–26], nearly all participants in this study responded that there were more male leaders in surgical departments that were rotated in or contacted (96.27%). This indicated that there may be a lack of female role models in the current surgical field. A survey conducted in Canada [23] reported that medical students have a greater proportion of male surgical role models, and that male students were more likely to be influenced by role models and choose surgical careers than female students. As the lack of female role models has often been cited as a key factor preventing female students from entering the surgical field, across contexts [27–29], the lack of female role models in China may be an obstacle to the careers of female surgeons and potential female surgeons. Few respondents reported gender barriers in recruitment, accounting for only 32.19% of the respondents; however, this may constitute an underestimation, because the respondents had received only an undergraduate education and as yet lacked experience of recruitment. Their understanding of recruitment may then have come from others’ experiences, which may have ignored gender perspectives. Among the respondents who reported that male leaders dominated and noted gender barriers in recruitment and who nevertheless chose to pursue a career in surgery, there were more male than female respondents. In the current, male-dominated surgical field, male students are more likely to receive preferential treatment for entry opportunities and career prospects, which, to a certain extent, increases the advantage for males to choose a surgical career. The self-cognition of medical students will have an impact on their career choices. Compared to male respondents, more female respondents thought that females were not suitable for surgical careers, which reduced their willingness to engage in a surgical career. Physiological factors such as high labor intensity was one of the primary reasons females were seen as unsuitable for surgical occupations. The actual dominance of males in the surgical field only serves to reinforce this viewpoint. In addition, females who had witnessed gender barriers to surgical career recruitment were also less likely to pursue a career in surgery, though non-significantly.

It is necessary to take correct measures to reduce or eliminate the influence of gender bias on career choices. This includes ensuring equal opportunities for females to pursue graduate education in surgery, enter the field of surgery, and have careers in surgery. Realizing equality of opportunity is not an easy task; it will require both education and labor departments to introduce appropriate policies and regulatory measures. In addition, it may be possible to eliminate the “gender fit” view in the field of surgery by providing more female role models. Increasing the possibility of cooperation with surgical tutors and providing more surgical training opportunities can also increase females’ willingness to engage in surgery.

### Limitations

The study has an important limitation: it only examined whether respondents had a willingness to pursue a surgical career, and did not classify the degree of their desire. We believe that although this survey does not reflect the degree of willingness to pursue a surgical career, it can demonstrate the likelihood of medical students pursuing surgical careers.

## Conclusion

Medical students are more optimistic about their willingness to pursue a career in surgery. Interest is the main driver for our participating medical students to choose a career in surgery. A male–female gender gap in preference for surgical careers exists among Chinese medical students. Gender bias reflected by recruitment barriers and dominance of male leadership affect medical students’ choice of surgical careers by gender. To promote the engagement of females in the field of surgery, it is necessary to eliminate the negative effects of gender prejudice by ensuring equal opportunities for higher education and employment in surgery while also promoting more female role models.

## Data Availability

The datasets generated and analysed during the current study are not publicly available due public availability of data would compromise confidentiality and privacy of participants but are available from the corresponding author on reasonable request.
